# Care Robotics: An Assessment of Professional Perception in the Face of the COVID-19 Pandemic

**DOI:** 10.3390/healthcare11070946

**Published:** 2023-03-24

**Authors:** Alexandra González Aguña, Blanca Gonzalo de Diego, Sandra Páez Ramos, Marta Fernández Batalla, María Lourdes Jiménez Rodríguez, José María Santamaría García

**Affiliations:** 1 Henares University Hospital, Community of Madrid Health Service (SERMAS), 28822 Madrid, Spain; 2 Research Group MISKC, Department of Computer Science, University of Alcala, Polytechnic Building, University Campus, Barcelona Road Km. 33.6, 28805 Alcalá de Henares, Spain; blanca94gd@gmail.com (B.G.d.D.); sandra.pr17@gmail.com (S.P.R.); marta.fdezbatalla@gmail.com (M.F.B.); lou.jimenez@uah.es (M.L.J.R.); chesantgar@hotmail.com (J.M.S.G.); 3 Meco Health Centre, Community of Madrid Health Service (SERMAS), 28880 Madrid, Spain; 4 Computer Science Department, University of Alcala, 28805 Madrid, Spain

**Keywords:** health informatics, robotics, COVID-19, assistive technologies, health technology assessment

## Abstract

The COVID-19 crisis accelerated the adoption of technologies. Technological advancement is also expected in robotics applied to any sector, including in healthcare. The aim is to assess the professional perception of care robotics facing COVID-19. This study aimed to (1) select a tool for assessing different aspects of healthcare, (2) analyse the professional perception about the development, usefulness and helpfulness of technologies and robotics in the field of healthcare and (3) evaluate the correlation between the perceived helpfulness of care robotics and the selected tool. We implement five validated clinical tests which integrate 80 items about a person and their clinical situation. From the sample of 46 professionals, 95.65% affirmed that technology was moderately to completely useful for professional performance in the context of the pandemic, lowering to 67.39% when asked only about robotics; 93.48% stated that the inclusion of robotics in at least one health area affected by COVID-19 would have helped them. Finally, the variables extracted from clinical tests corresponded to the most relevant health areas as identified by the professionals. This research shows the potential of care robotics oriented towards healthcare from a care paradigm.

## 1. Introduction

Almost three years after the first cases of SARS-CoV-2 coronavirus infection were identified, the World Health Organization (WHO) now counts more than 635 million people diagnosed with COVID-19 and more than 6.5 million deaths [1], all this in the context of a global crisis with health as its epicentre, but with a socio-economic impact on the entire planet [2,3].

People diagnosed with COVID-19 may experience a multitude of symptoms. It is a clinical process with a wide range of degrees of severity, from asymptomatic cases to extremely severe cases leading to death, and on top of that, it affects people of any age and vulnerability, although with a special impact on the most fragile people with advanced age, a weak immune system and other concomitant chronic diseases [4]. In addition, preventive measures to avoid the spread of infection included periods of individual and collective isolation. During the worst months of the COVID-19 pandemic, when vaccination was not yet available and health systems were in a critical situation, many regions declared citywide and countrywide containment rules [5]. These living conditions changes, along with the fear of infection, had an impact on mental health that was later visible in the increase in cases of anxiety and depression, as well as other associated disorders [6].

Ultimately, COVID-19 represents a health process that impacts a person physically, psychologically and socially [7]. In this sense, healthcare requires models that analyse each situation from a perspective centred on the person and their vulnerability, along with the risk conditions of the environment in which they live and work. The *Knowledge Model about Person Care* integrates this perspective of analysing the person and their environment in order to subsequently analyse their clinical situation, possible health outcomes and the most appropriate interventions [8]. A triangulation analysis allows one to identify the main care needs and to monitor the evolution of health outcomes [9,10].

The United Nations (UN) published a report on the status of the Sustainable Development Goals in 2022 after the impact of crises such as COVID-19, an impact that is evident in Goal 3: Good health and well-being, but also in the other ones where technologies are highlighted, such as in Goal 9: Industry, innovation and infrastructure [11,12].

The pandemic demonstrated the importance of industrialisation, technological innovation and resilient infrastructures [7]. More specifically, during the global crisis, digital health technologies have proven to be fundamental tools. In fact, the UN recognizes that the crisis has accelerated the digitalization of many services such as the access to healthcare [12]. Thus, the adoption of technologies in areas such as industry, education, research and health has undergone an acceleration, and this is also expected to happen in the field of robotics [13]. 

Robotics has already been used for supply logistics, infection prevention and surveillance and population healthcare, and its use is expected to grow [14,15]. The International Federation of Robotics (IFR) predicts an increase in the demand for robots [16]. A robot is a “*programmed actuated mechanism with a degree of autonomy to perform locomotion, manipulation or positioning*”, while a robotic device is a “*mechanism developed with robotic technology, but not fulfilling all characteristics of a robot*” [17]. Among the different types of robots, the International Standard Organization (ISO) 8373:2021 Robotics–Vocabulary distinguished in 2021 between industrial robots, medical robots and service robots [17]. A framework such as ISO 13482:2014 specifies safety requirements for robots and personal care robotic devices, which serve to perform useful tasks (excluding automation applications) and are classified into three groups: mobile servant robot (“*to perform serving tasks in interaction with humans, such as handling objects or exchanging information*” [18]), physical assistant robot (“*to perform required tasks by providing supplementation or augmentation of personal capabilities*” [18]) and person carrier robot (“*with the purpose of transporting humans to an intended destination*” [18]). The functions and areas of implementation of these robots and robotic devices are evidenced by studies on experiences related to daily life tasks such as feeding, hygiene or ambulation, as well as to improve communication.

In the context of COVID-19, the European Commission launched the “*Join the AI-ROBOTICS versus COVID-19*” initiative of the European Artificial Intelligence (AI) Alliance with the aim of gathering deployable solutions from the field of AI and robotics to address the various problems that emerged as a consequence of the global crisis [19].

Currently, the different publications on the use of robotics in the field of healthcare in relation to COVID-19 focus on its use to maintain social distance between healthcare professionals and people with a suspected or diagnosed illness, the collection of data and samples for diagnosis or its usefulness in area disinfection tasks. Likewise, some research has delved into the perspective of professionals and patients in relation to the use of robotics in healthcare and, more specifically, in relation to COVID-19. [20,21,22,23,24,25,26,27,28].

However, robotics can be used for more aspects of healthcare than what was already developed prior to COVID-19 and would promote a comprehensive care that allows for the assessment and treatment of all person’s needs. In addition, robotics cuts across multiple disciplines and the perspective of different professionals should be integrated because it is currently cross-disciplinary.

In this sense, this research asks about the perception of the different professional branches with regard to robotics within the framework of technologies and, more specifically, about care robotics to attend a person’s needs derived from the COVID-19 health process.

### Main Aim

The main aim of the study was to assess the professional perception of care robotics in the face of COVID-19. This study aimed to: (1) select a tool for assessing different aspects of healthcare; (2) analyse the professional perception about the development, usefulness and helpfulness of technologies and robotics in the field of healthcare; and (3) evaluate the correlation between the perceived helpfulness of care robotics and the selected tool. 

## 2. Materials and Methods

### 2.1. Design

**Figure 1:**
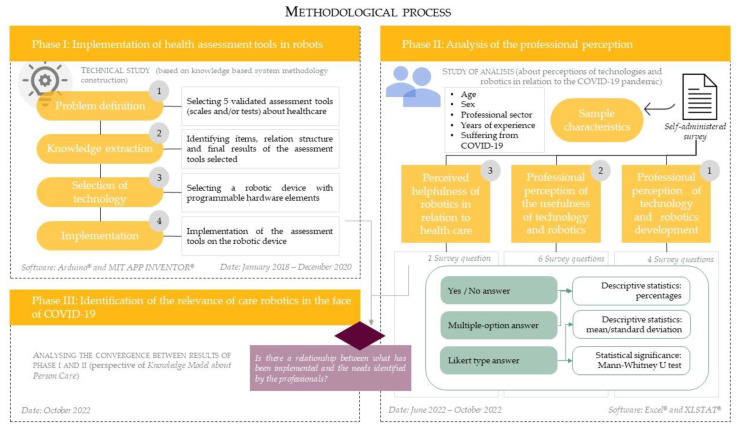
Methodological process scheme.

This study was conducted following three steps (Figure 1):

1) Selecting a tool for assessing different aspects of healthcare.
2) Analysing the professional perception about the development, usefulness and helpfulness of technologies and robotics in the field of healthcare in relation with COVID-19.
3) Clarifying the correlation between the perceived helpfulness of care robotics and the selected tool.

### 2.2. Procedure

The procedure is described below for each of the three phases and includes the main characteristics of each study and the corresponding data analysis.

#### 2.2.1. Phase I: Selecting and Implementing Health Assessment Tools in Robots

The first phase started in January 2018, ended in December 2020 and consisted of a technical study of the implementation of health assessment tools in a robotic device. The methodology consisted of the following sequence:

- Problem definition: First, the researchers selected five assessment tools (scales and/or tests) that captured different areas of a person’s healthcare. These assessment tools had to be validated scales and/or tests that could be used to analyse the person and different areas of their health situation.
- Knowledge extraction: once the assessment tools were selected, the next step was to identify the items (endpoints), the relational structure of the test and the possible final results.
- Selection of technology for implementation: A robotic device connected to a mobile phone (smartphone or tablet) was built for support or assistance during interaction. The programmable hardware elements of the robotic device included an Arduino Mega 2560 microcontroller, a Bluetooth module HC-06, a micro-SD (storage data) module, liquid crystal display (LCD) with interintegrated circuit (I2C) connection module and an RGB (red, green, blue) light-emitting diode.
- Implementation: Assessment tools were implemented on the robotic device by using the Arduino^®^ development environment [29] version 1.8.8. and the supporting mobile application using MIT APP INVENTOR^®^ software [30].

#### 2.2.2. Phase II: Analysis of the Professional Perception

The second phase ran from June to October 2022 during which we conducted a study analysing the professional perceptions of some aspects of technology and robotics in relation to their involvement during the COVID-19 pandemic.

The design employed a convenience sampling during the “Day of the Professions” event held in Madrid (Spain) on 22 September 2022. This event is an open event for professional associations that aims to bring professions closer to the public and to provide guidance to high-school and university students [31]. The selection criteria included all professionals participating from the different professional associations. Citizens and students visiting the event were not part of the sample. 

Data were collected by means of a self-administered survey, titled “Perception of robotics by different professional sectors”, which was designed specifically for this research. The survey is available in Supplementary Materials S1. The survey included six questions on the characteristics of the sample of participants and eleven questions to assess three aspects or themes:

- Sample characteristics: data on the profile of the participants, including age, sex, professional sector, years of experience and whether they suffered from COVID-19.
- Professional perception of technology and robotics development: a subjective assessment by the participants of the progress or improvement shown by technology and robotics in their professional field.
- Professional perception of the usefulness of technology and robotics: the participants’ subjective assessment of the benefit or interest achieved by technology and robotics in their professional field.
- Perceived helpfulness of robotics in relation to healthcare: A participant’s subjective assessment of the assistance or cooperation offered by robotics in the area of healthcare needs. This question was used for Phase III of the research.

The classification of the questions in the thematic areas is summarised in Table 1.

All participants were informed about the purpose of the research, the anonymous and voluntary nature of the survey and about the ability to refuse to participate or request to cancel their participation at any time, without affecting their participation in the rest of the services offered during the event. 

Data were collected in paper format and then transferred to a spreadsheet in Microsoft Excel^®^ by an independent researcher who also analysed the responses. The analysis included descriptive statistics for Likert type responses and percentages for the rest of the questions.

To study significance, the Mann–Whitney U test was applied to compare the different types of participants according to characteristics (independent variables) and the responses obtained from those questions whose evaluation was in Likert format. Calculations were performed using Microsoft Excel^®^ (Microsoft, Redmond, WA, USA) and XLSTAT^®^ software (Lumivero, Denver, CO, USA).

#### 2.2.3. Phase III: Identification of the Relevance of Care Robotics in the Face of COVID-19

The third phase was conducted in October 2022. This phase identified the relevance of care robotics to the COVID-19 pandemic by analysing the convergence between the needs assessed by implemented tools in the robotic device (Phase I) and the needs identified by the professionals in question 13 of the survey (Phase II).

In addition, this analysis included a perspective from the *Knowledge Model about Person Care* to determine whether the assessment tasks performed by the implemented tools managed to cover the different phases of the healthcare process [8].

## 3. Results

The results are shown below according to the stages of the procedure:

### 3.1. Phase I: Selecting and Implementing Health Assessment Tools in Robots

In total, five person-centred healthcare assessment tools were selected. The summary of the selection is shown in Table 2.

**Figure 2:**
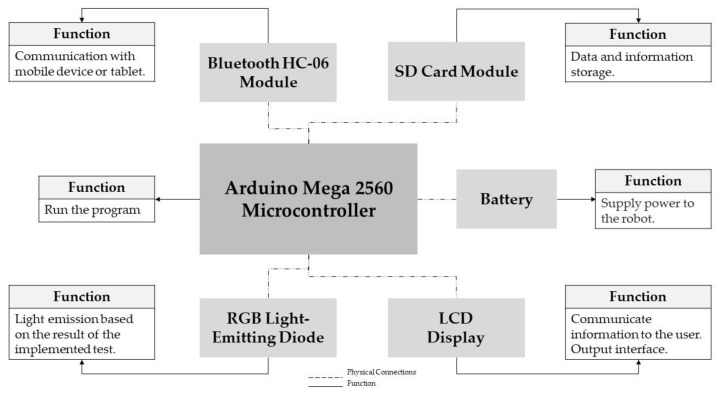
Robot hardware diagram and its functions.

These assessment tools were implemented in a robotic device. The hardware elements used for this device and the functions of each of the elements are shown in the scheme in Figure 2. 

The implementation of the five assessment tools in the device used the Arduino and MIT App Inventor^®^ development environments. The corresponding programming codes, connection schema and some photos are fully available in the Supplementary Materials S2.

### 3.2. Phase II: Analysis of the Professional Perception

#### 3.2.1. Participant Profile

The total sample included 46 participants, with a mean age of 43.67 years, a range of 21–62 years, and approximately two thirds were female. Regarding their professional sector, most of them worked in the healthcare sector. As for their academic training, more than half of them did not have a health-related qualification. Some participants worked in the healthcare sector, but their academic background was from another sector. More than eighty percent of the participants had more than ten years of professional experience.

Regarding their COVID-19 history, the majority of participants (73.91%) claimed to have had the disease. The figure was reduced to 43.48% when asked about family members diagnosed with COVID-19 in the past, and 26.09% of participants claimed that both they and a family member had had the disease.

A summary of the characteristics of the participants is shown in Table 3.

#### 3.2.2. Professionals’ Assessment of Technology and Robotics

- Professional perception of technology and robotics development.

The results of the questions on technological development and robotics according to the total number of participants by professional sector are shown in Table 4.

The analysis of centrality and dispersion on the Likert type responses (Q6, Q7) is shown in Table 5.

The results showed a majority with an average score higher than three points. Only three scores on question Q7 about the perception of the development of robotics in their professional sector had an average score between two and three points and a standard deviation (SD) higher than one point. In this sense, the SD showed a range between 0.55, for question Q6 on the perception of technological development in the professional sector in the category of professional experience <5 years, and 1.53 for Q7 on the perception of robotics development in the professional sector in the group with a professional experience of 5–10 years. The results highlighted that the median and the mode decreased for Q6 with respect to Q7. The median in Q7 in the group with a professional experience of 5–10 years was less than three. In all other cases, the median and the mode were higher than three points.

The statistical significance of these categories of analysis is shown in Table 6 comparing the different characteristics of the sample of participants. 

The results showed a statistical significance (*p* < 0.0001) for question 6 (on the perception of technological development in their professional sector) comparing the group of architects and engineers versus healthcare professionals and the group with <10 years versus the group with >10 years of professional experience. In addition, question 7 about the perception of robotics development showed a statistical significance with *p* = 0.036 when comparing the architects and engineers’ group versus the legal and economic group.

- Professional perception of the usefulness of technology and robotics.

The results from the questions about the usefulness of technology and robotics by total number of participants and by professional sector are shown in Table 7.

The analysis of centrality and dispersion of the Likert type responses (Q8, Q9, Q10, Q17) is shown in Table 8 and Table 9.

The results showed a majority with an average score higher than three points. Only one score in question Q10 about the usefulness of robotics if they had used it had an average score between two and three points and a standard deviation (SD) higher than one point. The SD showed a range between 0.58 for Q9 and Q17 in the category of professional experience of 5–10 years and 1.73 for question Q10 for the legal and economic professional sector. The median and the mode in Q8, Q9, Q10 and Q17 were higher than three points.

The statistical significance of these categories of analysis is shown in Table 10 comparing the different characteristics of the sample of participants.

The results showed a statistical significance (*p* < 0.005) for question 8 on the tendency to use robotics when comparing the group of architects and engineers versus the legal and economic sector.

- Perceived helpfulness of robotics in relation to healthcare.

The results of question 13 (Q13) of the survey (on the inclusion of robotics for healthcare needs related to a COVID-19 infection) are shown in Table 11.

Overall, 93.48% of the sample stated that the inclusion of robotics would help in at least one area of health affected by COVID-19. Breathing, mobility and communication and interpersonal relationships were selected by more than 50% of the sample.

Professionals who had not suffered from COVID-19 highlighted the helpfulness in aspects related to breathing (72.73%) compared to professionals who had suffered COVID-19, where approximately 50% responded with aspects related to breathing, mobility and communication and interpersonal relationships. Approximately 68.42% of the participants with a university education in health sciences highlighted the aspects related to communication and interpersonal relationships.

The responses of this question were used to elaborate the next phase of this study.

### 3.3. Phase III: Identification of the Relevance of Care Robotics in the Face of COVID-19 

Finally, the third phase of the study related the results achieved by implementing the health assessment tools in a robotic device and the results obtained for question 13 (Q13) of the survey about the perceived helpfulness of robotics in relation to healthcare.

**Figure 3:**
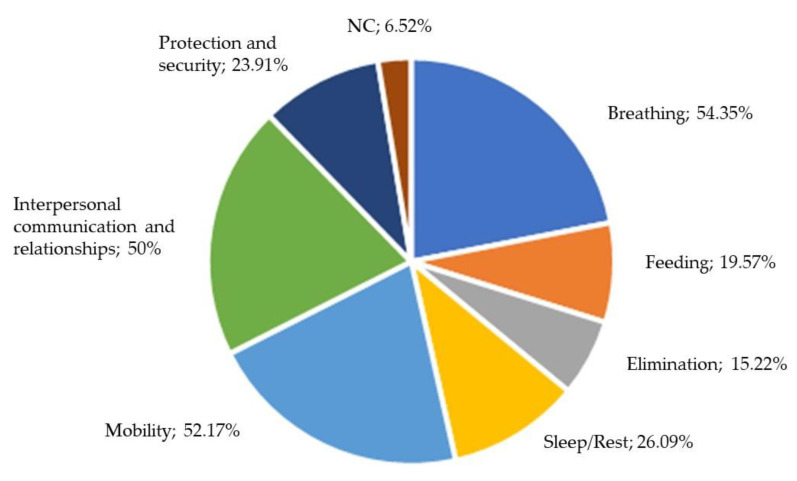
Results (Q13) on health aspects where robotics can help (percentages are shown each one about the total of participants).

Participants claimed that robotics could help with breathing, mobility and interpersonal communication and relationships. Figure 3 shows the results to question Q13 of the survey.

The aspects related to breathing were collected with the robotic device by means of the COPD Test (CAT) assessment tool. Aspects related to safety protection and mobility were related to the Morisky Medication Adherence Scale and the Fall Risk Index because these tools assess people’s safety with the adherence to drug treatment and risk of falls. Moreover, both are related because some types of drugs (sedatives, diuretics, antihypertensives, antiparkinsonian drugs and antidepressants) are risk factors for falls. Likewise, aspects related to communication and relationships between people were represented in the robotic device through the implementation of the State-Trait Anxiety Inventory for Children (STAIC), which assesses the state and trait anxiety of a children linked to the way they relate and communicate with their environment.

The study implemented the Care Vulnerability Index tool in the robotic device. This tool assesses the characteristics of people in relation to their environment to determine their vulnerability. This contribution integrated a person-centred model perspective because the robotic device allowed us to start any health assessment by determining the overall vulnerability of the person, adding later all the assessments according to age (STAIC for children and Fall Risk Index for the elderly), health problems (CAT), human responses such as anxiety (STAIC) or the monitoring of health interventions in the presence of a health problem with a prescribed pharmacological treatment (Morisky Medication Adherence Scale).

## 4. Discussion

Similar studies have concentrated the objective of their analysis on the perception of technologies or robots in samples of health professionals and patients, both potential end users of these devices [20,21,22,23,24,25,26,27,28,37,38,39,40].

The most similar study was published by Savela, Turja and Oksanen [41], who carried out a systematic literature review about the acceptance of robots in different professional sectors. Their research showed that the attitude of professionals in the health and social sector was better towards robots than in other sectors, and people without previous experience had a worse attitude more often. 

Other studies such as that by Morone et al. [42] focused on the perception of the professional about the integration of robotics in rehabilitation and care activity and on the impact of ethics, their concerns and suggestions. Gillisen et al. [43] studied medical students’ attitudes towards AI and other digital tools, including robotics, agreeing with their concerns about the replacement of humans by robots.

Thus, regarding the methodology of this study, the design responded to the need to carry out different but independent phases. Each phase had a different purpose and a development framework, ranging from the technological field, with the development and implementation of assessment tools in a robotic device, to the field of social and health sciences with survey-based designs. According to previous literature review studies, researchers prefer questionnaire-based designs (around 80% of the literature reviewed and interviews). Hardly any studies were found on real-time, real user experience of the process [44,45].

According to the results, the assessment tools selected and implemented covered all aspects of healthcare according to current regulations and strategies. In this sense, vulnerability and its evolution towards frailty have been terms of interest in recent years. The WHO works on these concepts by associating them with the conditions of populations and, fundamentally, with the socio-economic conditions of the environment [46,47]. A similar perspective is the one applied in Spain, where a vulnerability analysis is established according to populations and the conditions of the neighbourhoods where they live [48]. The rest of the assessment tools implemented in the robotic device were internationally known and validated in previous published studies [32,33,34,35,36]. In Spain, these tools are used by the public health system and are integrated in the Primary Care Services Portfolio, a document that includes the areas of action of health professionals [49]. 

On the perception of the development and use of technology and robotics, the studies show two lines of work: personal care robots are assigned to tasks such as assisting the person with feeding, personal hygiene, monitoring vital signs (especially associated with respiratory infection); or person-carrying robots in the mobilisation aid to improve personal autonomy or to collaborate with health professionals in tasks that require a physical effort [20,50,51,52,53,54,55]. During the COVID-19 pandemic, these technologies were applied to prevent infection by allowing tasks to be performed while maintaining social distance between professionals and people with suspected or diagnosed COVID-19. For example, robots were able to take biological samples for diagnostics, monitor vital signs, monitor compliance with containment measures or disinfect areas [16,21].

Technologies and robotics were also used for communication during this period. The WHO offered a chatbot to keep the population informed and resolve doubts in the face of increasing misinformation in society and the media [56]. During the days of isolation, people used technology to maintain communication and social relationships through video calls or online group activities.

Technologies and robotics increased their presence in society and especially in the areas of healthcare that were highlighted by the survey participants. A study conducted in Massachusetts in 2020 found an increase (perceived usefulness in hospital settings vs. perceived usefulness in hospital settings during COVID-19 pandemic) in the mean number of people who perceived the use of robots as acceptable, for tasks such as telehealth interviewing or obtaining nasal swabs [39].

### Limitations and Future Lines

Some limitations of the study should be noted in order to understand the scope of this research and its future directions.

Firstly, the assessment tools implemented were scarce compared to the large volume of validated scales and indices that currently exist. The study demonstrated that their implementation was possible, but this sample should be increased in the future to achieve a detailed and flexible global assessment for use in any situation. The robotic device used had hardware and software limitations. Current developments make it possible to technically improve this device and to integrate other functionalities in the future.

A type of robot that was not analysed in this study is social robots used for companionship. These robots can have human or animal appearance and focus on simulating behaviour (including human emotions) to enhance well-being through the feeling of companionship. However, this area of research must address cultural, social and political factors that contextualise the interaction process and personalise the meaning of verbal and nonverbal language [23,40,57].

In addition, any future research requires an analysis on the ethical implications of the use of robotics for healthcare, a critical aspect that requires paradigms and models of knowledge about what care is and how people take care of themselves throughout their lives in any environment and situation.

## 5. Conclusions

Care robotics, a branch of care-oriented robotics, has been established as a part of artificial intelligence that integrates mechanical engineering, electronic engineering and computer science for the design of automated systems that perform tasks and even simulate human behaviour.

This research showed the development potential of care robotics by verifying that it was possible to implement clinical assessment tools in a robotic device. Furthermore, a favourable assessment was obtained regarding the development, usefulness and helpfulness of this technology, particularly healthcare-oriented robotics.

In conclusion, research and progress in the field of robotics for persons’ care were proposed with an approach based on paradigms that integrated the understanding of the person and their environment: how they live, how they take care of themselves and what tools can be applied for healthcare promotion, prevention and therapeutic treatment. 

## Figures and Tables

**Table 1:** Survey question classification.

Category	Type of Answer	Survey Questions
Characteristics of the sample of participants	-	1, 2, 3, 4, 5, 11
Professional perception of technology and robotics development	Likert type Yes/No Multiple option	6, 7 15 16
Professional perception of the usefulness of technology and robotics	Likert type Yes/No Multiple option	8, 9, 10, 17 12 14
Professional perception of the help obtained from robotics in healthcare	Multiple option	13

**Table 2:** Selected assessment tools, purpose and number of items.

Assessment Tools	Purpose	Number of Items	Reference
Care Vulnerability Index	Assessment of a person’s vulnerability	12	[32]
State-Trait Anxiety Inventory for Children (STAIC)	Assessment of anxiety in children (based on an STAI survey for adolescents and adults)	40	[33]
The COPD Assessment Test (CAT)	Assessment of the impact of chronic obstructive pulmonary disease (COPD) on quality of life	8	[34]
Morisky Medication Adherence Scale	Assessment of adherence to medication treatment	4	[35]
Downton Fall Risk Index	Assessment of risk factors for falls	16	[36]

**Table 3:** Characteristics of participants.

Characteristics		*n* (%) (N = 46)
Sex	Male	15 (32.61%)
	Female	30 (65.22%)
	No answer	1 (2.17%)
Professional sector	Architecture and engineering	8 (17.39%)
	Law and economics	10 (21.74%)
	Health sciences	26 (56.52%)
	No answer	2 (4.35%)
University degree sector	Health sciences	19 (41.30%)
	Not health sciences	24 (52.17%)
	No answer	3 (6.52%)
Professional experience	<5 years	6 (13.04%)
	5–10 years	3 (6.52%)
	>10 years	37 (80.43%)
Participant had COVID-19	Yes	34 (73.91%)
	No	11 (23.91%)
	No answer	1 (2.17%)
Relatives of the participant had COVID-19	Yes	20 (43.48%)
	No	25 (54.35%)
	No answer	1 (2.17%)

**Table 4:** Distribution of responses on technological development and robotics.

Question	Total		A and E		L and E		H. Sc.	
	*n*	%	*n*	%	*n*	%	*n*	%
Q6. In recent years you have noticed developments in aspects related to technology applied to the professional sector in which you work:								
I have been able to observe an extensive development of technology.	23	50.00	5	62.50	5	50.00	12	46.15
I have been able to observe a substantial development of technology.	18	39.13	2	25.00	4	40.00	11	42.31
I have been able to observe a moderate development of technology.	4	8.70	1	12.50	1	10.00	2	7.69
I have been able to observe a scarce development of technology.	1	2.17	0	0.00	0	0.00	1	3.85
I have not been able to observe any development of technology.	0	0.00	0	0.00	0	0.00	0	0.00
Q7. In recent years you have noticed developments in aspects related to robotics applied to the professional sector in which you work:								
I have been able to observe an extensive development of robotics.	1	2.17	1	12.50	0	0.00	0	0.00
I have been able to observe a substantial development in robotics.	19	41.30	5	62.50	1	10.00	12	46.15
I have been able to observe a moderate development of robotics.	13	28.26	0	0.00	5	50.00	8	30.77
I have observed little development in robotics.	6	13.04	1	12.50	1	10.00	3	11.54
I could not observe any robotics development.	7	15.22	1	12.50	3	30.00	3	11.54
Q15. Do you think your profession is positioned to take an active role in the development of technological tools, including robotics?								
Yes	31	67.39	8	100.00	2	20.00	19	73.08
No	14	30.43	0	0.00	8	80.00	6	23.08
No answer	1	2.17	0	0.00	0	0.00	1	3.85
Q16. If your answer above was yes, what role do you think your profession can play in the field of robotics?								
Robot development: programming and construction.	9	19.57	4	50.00	0	0.00	5	19.23
Implementation of robots in different real-world environments.	11	23.91	3	37.50	0	0.00	8	30.77
Building knowledge models for robotic implementation.	12	26.09	2	25.00	2	20.00	7	26.92
Addressing ethical and legal aspects related to the implementation of robots.	10	21.74	2	25.00	2	20.00	5	19.23
Synergic team management: Project management	9	19.57	3	37.50	0	0.00	6	23.08
No answer	13	28.26	0	0.00	6	60.00	7	26.92

Two participants did not answer the question about the professional sector and for this reason, the total is greater than the sum of the categories in some questions. A and E: architecture and engineering; L and E: law and economics; H. Sc: health sciences.

**Table 5:** Centrality and dispersion in Likert type questions 6 and 7.

Characteristics		Q6						Q7					
		$\overset{-}{X}$ (SD)	Me	Mo	Range	Min	Max	$\overset{-}{X}$ (SD)	Me	Mo	Range	Min	Max
Total		4.37 (0.74)	4.5	5	3	2	5	3.02 (1.13)	3	4	4	1	5
Sex	Male	4.20 (0.94)	4	5	3	2	5	3.33 (0.90)	3	4	3	2	5
	Female	4.43 (0.63)	4.5	5	2	3	5	2.93 (1.17)	3	4	3	1	4
Professional sector	A and E	4.50 (0.76)	5	5	2	3	5	3.50 (1.31)	4	4	4	1	5
	L and E	4.40 (0.70)	4.5	5	2	3	5	2.40 (1.07)	3	3	3	1	4
	H. Sc.	4.31 (0.79)	4	5	3	2	5	3.12 (1.03)	3	4	3	1	4
University degree Sector	H. Sc.	4.37 (0.68)	4	5	2	3	5	3.00 (1.11)	3	4	3	1	4
	Not H. Sc.	4.38 (0.82)	5	5	3	2	5	3.00 (1.22)	3	4	4	1	5
Professional experience	<5 years	4.50 (0.55)	4.5	5	1	4	5	3.33 (0.82)	3.5	4	2	2	4
	5–10 years	4.00 (1.00)	4	-	2	3	5	2.33 (1.53)	2	-	3	1	4
	>10 years	4.38 (0.76)	5	5	3	2	5	3.03 (1.14)	3	4	4	1	5
Participant had COVID-19	Yes	4.32 (0.77)	4	5	3	2	5	3.06 (1.15)	3	4	4	1	5
	No	4.45 (0.69)	5	5	2	3	5	2.91 (1.14)	3	3	3	1	4

A and E: architecture and engineering; L and E: law and economics; H. Sc.: health sciences; $\overset{-}{X}$: average; SD: standard deviation; Me: median; Mo: mode; Min: minimum; Max: maximum.

**Table 6:** Statistical significance analysis about technological development and robotics.

Characteristics	Related Samples	Q6 (*p*-Value)	Q7 (*p*-Value)
Sex	Male	0.551	0.4
	Female		
Professional Sector	Architecture and engineering	0.896	0.036
	Law and economics		
	Architecture and engineering	<0.0001	0.248
	Health sciences		
	Law and economics	0.799	0.068
	Health sciences		
University degree sector	Health professional	0.846	0.949
	Not health professional		
Professional experience	<10 years	<0.0001	0.965
	>10 years		
Participant had COVID-19	Yes	0.705	0.692
	No		
Relatives of the participant had COVID-19	Yes	0.637	0.421
	No		

**Table 7:** Distribution of responses on the usefulness of technology and robotics.

Question	Total		A and E		L and E		H. Sc.	
	*n*	%	*n*	%	*n*	%	*n*	%
Q8. What do you think will be the trend in the use of robotics in your professional sector in the coming years?								
I think the use of robotics in my sector will be extensive.	14	30.43	2	25.00	1	10.00	10	38.46
I think the use of robotics in my sector will be substantial	10	21.74	4	50.00	0	0.00	6	23.08
I think the use of robotics in my sector will be moderate.	15	32.61	2	25.00	8	80.00	4	15.38
I think the use of robotics in my sector will be low.	7	15.22	0	0.00	1	10.00	6	23.08
I think the use of robotics in my sector will be nil.	0	0.00	0	0.00	0	0.00	0	0.00
Q9. The last two years have been marked by the COVID-19 pandemic. Regarding the use of technology in professional performance in this context of health emergency:								
The use of technology in professional performance has been entirely useful in the context of the COVID-19 pandemic.	32	69.57	5	62.50	8	80.00	17	65.38
The use of technology in professional performance has been substantially useful in the context of the COVID-19 pandemic.	8	17.39	1	12.50	1	10.00	6	23.08
The use of technology in professional performance has been moderately useful in the context of the COVID-19 pandemic.	4	8.70	2	25.00	1	10.00	1	3.85
The use of technology in professional performance has been only moderately useful in the context of the COVID-19 pandemic.	1	2.17	0	0.00	0	0.00	1	3.85
The use of technology in professional performance has not been useful in the context of the COVID-19 pandemic.	1	2.17	0	0.00	0	0.00	1	3.85
Q10. And have you found the use of robotics useful if you have used it?								
The use of robotics in professional performance has been entirely useful in the context of the COVID-19 pandemic.	12	26.09	2	25.00	2	20.00	7	26.92
The use of robotics in professional performance has been substantially useful in the context of the COVID-19 pandemic.	8	17.39	1	12.50	1	10.00	6	23.08
The use of robotics in professional performance has been moderately useful in the context of the COVID-19 pandemic.	11	23.91	4	50.00	1	10.00	5	19.23
The use of robotics in professional performance has been only marginally useful in the context of the COVID-19 pandemic.	3	6.52	1	12.50	1	10.00	1	3.85
The use of robotics in professional performance has not been useful in the context of the COVID-19 pandemic.	7	15.22	0	0.00	2	20.00	5	19.23
No answer	5	10.87	0	0.00	3	30.00	2	7.69
Q12. In light of this personal experience, do you think that the COVID-19 pandemic has generated new needs and demands in the population that position technological tools as a plausible solution to address them?								
Yes	44	95.65	8	100	10	100	24	92.31
No	1	2.17	0	0.00	0	0.00	1	3.85
No answer	1	2.17	0	0.00	0	0.00	1	3.85
Q14. Which robotic devices do you think would have been useful for them? You can tick one or more.								
Robots for monitoring symptoms of COVID-19 infection.	23	50.00	6	75.00	4	40.00	12	46.15
Accompanying robots.	16	34.78	2	25.00	3	30.00	11	42.31
Robots to facilitate the performance of activities of daily living such as feeding, hygiene, dispensing medication, mobilisation, etc.	25	54.35	3	37.50	5	50.00	16	61.54
Educational robots for learning.	15	32.61	3	37.50	2	20.00	9	34.62
No answer	3	6.52	0	0.00	2	20.00	1	3.85
Q17. Regarding the following statement: Synergy between professions is useful in the creation and development of robots aimed at aiding and/or assisting human care.								
I fully agree.	23	50.00	4	50.00	5	50.00	13	50.00
I agree	17	36.96	2	25.00	4	40.00	10	38.46
I neither agree nor disagree	3	6.52	2	25.00	1	10.00	0	0.00
I disagree	2	4.35	0	0.00	0	0.00	2	7.69
I strongly disagree	0	0.00	0	0.00	0	0.00	0	0.00
No answer	1	2.17	0	0.00	0	0.00	1	3.85

A and E: architecture and engineering; L and E: law and economics; H. Sc.: health sciences.

**Table 8:** Centrality and dispersion of the Likert type questions 8 and 9.

Characteristics		Q8						Q9					
		$\overset{-}{X}$ (SD)	Me	Mo	Range	Min	Max	$\overset{-}{X}$ (SD)	Me	Mo	Range	Min	Max
Total		3.67 (1.08)	4	3	3	2	5	4.50 (0.91)	5	5	4	1	5
Sex	Male	3.67 (1.05)	4	3	3	2	5	4.67 (0.62)	5	5	2	3	5
	Female	3.67 (1.12)	3.5	3	3	2	5	4.40 (1.04)	5	5	4	1	5
Professional sector	A and E	4.00 (0.76)	4	4	2	3	5	4.38 (0.92)	5	5	2	3	5
	L and E	3.10 (0.74)	3	3	3	2	5	4.70 (0.67)	5	5	2	3	5
	H. Sc.	3.77 (1.21)	4	5	3	2	5	4.42 (1.03)	5	5	4	1	5
University degree sector	H. Sc.	3.79 (1.18)	4	5	3	2	5	4.42 (1.02)	5	5	4	1	5
	Not H. Sc.	3.54 (0.93)	3	3	3	2	5	4.63 (0.71)	5	5	2	3	5
Professional experience	<5 years	4.33 (1.03)	5	5	2	3	5	4.17 (1.17)	4.5	5	3	2	5
	5–10 years	3.33 (1.53)	3	-	3	2	5	4.67 (0.58)	5	5	1	4	5
	>10 years	3.59 (1.04)	4	3	3	2	5	4.54 (0.90)	5	5	4	1	5
Participant had COVID-19	Yes	3.79 (1.09)	4	5	3	2	5	4.65 (0.69)	5	5	3	2	5
	No	3.27 (1.01)	3	3	3	2	5	4.00 (1.34)	5	5	4	1	5

A and E: architecture and engineering; L and E: law and economics; H. Sc.: health sciences; $\overset{-}{X}$: average; SD: standard deviation; Me: median; Mo: mode; Min: minimum; Max: maximum.

**Table 9:** Centrality and dispersion of Likert type questions 10 and 17.

Characteristics		Q10						Q17					
		$\overset{-}{X}$ (SD)	Me	Mo	Range	Min	Max	$\overset{-}{X}$ (SD)	Me	Mo	Range	Min	Max
Total		3.37 (1.43)	3	5	4	1	5	4.36 (0.80)	5	5	3	2	5
Sex	Male	3.69 (1.32)	4	5	4	1	5	4.53 (0.64)	5	5	2	3	5
	Female	3.19 (1.49)	3	3	4	1	5	4.24 (0.87)	4	5	3	2	5
Professional sector	A and E	3.50 (1.07)	3	3	3	2	5	4.25 (0.89)	4.5	5	2	3	5
	L and E	3.00 (1.73)	3	5	4	1	5	4.4 (0.70)	4.5	5	2	3	5
	H. Sc.	3.38 (1.50)	4	5	4	1	5	4.36 (0.86)	5	5	3	2	5
University degree sector	H. Sc.	3.24 (1.52)	4	4	4	1	5	4.32 (0.95)	5	5	3	2	5
	Not H. Sc.	3.33 (1.39)	3	3	4	1	5	4.34 (0.71)	4	5	2	3	5
Professional experience	<5 years	3.83 (1.17)	4	5	3	2	5	4 (0.63)	4	4	2	3	5
	5–10 years	2.33 (1.53)	2	-	3	1	4	4.33 (0.58)	4	4	1	4	5
	>10 years	3.38 (1.45)	3	3	4	1	5	4.42 (0.84)	5	5	3	2	5
Participant had COVID-19	Yes	3.40 (1.43)	3.5	5	4	1	5	4.48 (0.71)	5	5	3	2	5
	No	3.20 (1.55)	3	3	4	1	5	4 (1.00)	4	4	3	2	5

A and E: architecture and engineering; L and E: law and economics; H. Sc.: health sciences; $\overset{-}{X}$: average; SD: standard deviation; Me: median; Mo: mode; Min: minimum; Max: maximum.

**Table 10:** Statistical significance analysis on the usefulness of technology and robotics.

Characteristics	Related Samples	Q8 (*p*-Value)	Q9 (*p*-Value)	Q10 (*p*-Value)	Q17 (*p*-Value)
Sex	Male	0.955	0.541	0.333	0.301
	Female				
Professional Sector	Architecture and engineering	0.005	0.588	0.608	0.908
	Law and economics				
	Architecture and engineering	0.812	0.791	0.734	0.767
	Health sciences				
	Law and economics	0.153	0.432	0.642	0.975
	Health sciences				
University degree sector	Health professional	0.429	0.507	0.938	0.781
	Not health professional				
Professional experience	<10 years	0.381	0.419	0.994	0.417
	>10 years				
Participant had COVID-19	Yes	0.147	0.129	0.742	0.138
	No				
Relatives of the participant had COVID-19	Yes	0.087	0.055	0.071	0.537
	No				

**Table 11:** Distribution of answers on the help of robotics in health care.

Question	Total		A and E		L and E		H. Sc.	
	*n*	%	*n*	%	*n*	%	*n*	%
Q13. Following on from the previous question, do you think that the inclusion of robotics in any aspects of your health affected by COVID-19 infection and its consequences would have helped you? You can tick one or several answers.								
Aspects related to breathing.	25	54.35	8	100	2	20.00	14	53.85
Aspects related to feeding.	9	19.57	1	12.50	1	10.00	7	26.92
Aspects related to elimination	7	15.22	2	25.00	0	0.00	5	19.23
Aspects related to sleep or rest	12	26.09	2	25.00	0	0.00	8	30.77
Aspects related mobility	24	52.17	5	62.50	4	40.00	14	53.85
Aspects related to communication and interpersonal relationships	23	50.00	2	25.00	6	60.00	15	57.69
Aspects related to safety and security	11	23.91	1	12.50	1	10.00	9	34.62
NC	3	6.52	0	0.00	1	10.00	2	7.69

A and E: architecture and engineering; L and E: law and economics; H. Sc.: health sciences.

## Data Availability

The data that support the findings of this study are available on request from the corresponding author.

## Supplementary Materials

The following supporting information can be downloaded at: https://www.mdpi.com/article/10.3390/healthcare11070946/s1, Supplementary Materials S1: Survey and Supplementary Materials S2: Programming Codes (https://github.com/BCareSpeculatrix/ProgrammingData.git accessed on 15 March 2023).
