# Supplementary material for: Care Robotics: An Assessment of Professional Perception in the Face of the COVID-19 Pandemic
Source: Healthcare (Basel). 2023 Mar 24;11(7):946. doi: 10.3390/healthcare11070946 (PMC10094221; doi:10.3390/healthcare11070946)
Supplement: Supplementary file 1 [file healthcare-11-00946-s001.zip › healthcare-2154264-supplementary.pdf]

SURVEY:

"*PERCEPTION OF ROBOTICS BY DIFFERENT PROFESSIONAL SECTORS*"

Q1. Age:_____

Q2. Sex:
- Male
- Female

Q3. Indicate the professional sector to which you are engaged:
- Professions in the Architecture and Engineering sector.
- Professions of the Legal and Economic sector.
- Professions in the Health Sciences sector.

Q4. Please, specify below the name of the profession to which you are dedicated:

____

Q5. How many years of experience in the professional sector do you have?:
- Less than five years.
- Between five and ten years.
- More than 10 years.

Q6. In recent years you have noticed developments in aspects related to technology applied to the professional sector in which you work:
I have been able to observe an extensive development of technology.
I have been able to observe a substantial development of technology.
I have been able to observe a moderate development of technology.
I have been able to observe a scarce development of technology.
I have not been able to observe any development of technology.

Q7. In recent years you have noticed developments in aspects related to robotics applied to the professional sector in which you work:
I have been able to observe an extensive development of robotics.
I have been able to observe a substantial development in robotics.
I have been able to observe a moderate development of robotics.
I have observed little development in robotics.
I could not observe any robotics development.

Q8. What do you think will be the trend in the use of robotics in your professional sector in the coming years?
I think the use of robotics in my sector will be extensive.
I think the use of robotics in my sector will be substantial
I think the use of robotics in my sector will be moderate.
I think the use of robotics in my sector will be low.
I think the use of robotics in my sector will be nil.

Q9. The last two years have been marked by the COVID-19 pandemic. Regarding the use of technology in professional performance in this context of health emergency:
The use of technology in professional performance has been entirely useful in the context of the COVID-19 pandemic.
The use of technology in professional performance has been substantially useful in the context of the COVID-19 pandemic.
The use of technology in professional performance has been moderately useful in the context of the COVID-19 pandemic.
The use of technology in professional performance has been only moderately useful in the context of the COVID-19 pandemic.
The use of technology in professional performance has not been useful in the context of the COVID-19 pandemic.

Q10. And have you found the use of robotics useful if you have used it?
The use of robotics in professional performance has been entirely useful in the context of the COVID-19 pandemic.
The use of robotics in professional performance has been substantially useful in the context of the COVID-19 pandemic.
The use of robotics in professional performance has been moderately useful in the context of the COVID-19 pandemic.
The use of robotics in professional performance has been only marginally useful in the context of the COVID-19 pandemic.
The use of robotics in professional performance has not been useful in the context of the COVID-19 pandemic.
No answer

Q11. COVID-19 pandemic has affected professional performance but above all our health and that of our loved ones, have you or any family member suffered from COVID-19?
Yes, I have had COVID-19.
Yes, some of my relatives have had COVID-19
Yes, both me and those close to me
No

Q12. In light of this personal experience, do you think that the COVID-19 pandemic has generated new needs and demands in the population that position technological tools as a plausible solution to address them?
Yes
No
No answer

Q13. Following on from the previous question, do you think that the inclusion of robotics in any aspects of your health affected by COVID-19 infection and its consequences would have helped you? You can tick one or several answers.
Aspects related to breathing.
Aspects related to feeding.
Aspects related to elimination
Aspects related to sleep or rest
Aspects related mobility
Aspects related to communication and interpersonal relationships
Aspects related to safety and security
NC

Q14. Which robotic devices do you think would have been useful for them? You can tick one or more.
Robots for monitoring symptoms of COVID-19 infection.
Accompanying robots.
Robots to facilitate the performance of activities of daily living such as feeding, hygiene, dispensing medication, mobilisation, etc.
Educational robots for learning.
No answer

Q15. Do you think your profession is positioned to take an active role in the development of technological tools, including robotics?
Yes
No
No answer

Q16. If your answer above was yes, what role do you think your profession can play in the field of robotics?
Robot development: programming and construction.
Implementation of robots in different real-world environments.
Building knowledge models for robotic implementation.
Addressing ethical and legal aspects related to the implementation of robots.
Synergic team management: Project management
No answer

Q17. Regarding the following statement: Synergy between professions is useful in the creation and development of robots aimed at aiding and/or assisting human care.
I fully agree.
I agree
I neither agree nor disagree
I disagree
I strongly disagree
No answer
